# Characterizing ‘health equity’ as a national health sector priority for maternal, newborn, and child health in Ethiopia

**DOI:** 10.1080/16549716.2020.1853386

**Published:** 2020-12-30

**Authors:** Nicole Bergen, Arne Ruckert, Lakew Abebe, Shifera Asfaw, Getachew Kiros, Abebe Mamo, Sudhakar Morankar, Manisha A. Kulkarni, Ronald Labonté

**Affiliations:** a Faculty of Health Sciences, Interdisciplinary School of Health Sciences, University of Ottawa, Ottawa, Canada; b Faculty of Medicine, School of Epidemiology and Public Health, University of Ottawa, Ottawa, Canada; c Faculty of Public Health, Institute of Health, Department of Health, Behavior and Society, Jimma University, Jimma, Ethiopia; d Faculty of Health Sciences, School of Nursing, University of Ottawa, Ottawa, Canada

**Keywords:** Ethiopia, health equity, health inequity, maternal, newborn and child health, policy analysis

## Abstract

**Background**: The pursuit of health equity is a priority in Ethiopia, especially with regards to maternal, newborn, and child health (MNCH). To date, there has been little characterization of the ‘problem’ of health inequity, and the normative assumptions implicit in the representation of the problem. Yet, such insights have implications for shaping the framing, incentivization, and implementation of health policies and their wider impact.

**Objective**: In this article, we characterize how health (in)equity is represented as a policy issue, how this representation came about, and the underlying assumptions.

**Methods**: We draw from Bacchi’s ‘what is the problem represented to be’ approach to explore how national-level actors in the health sector constitute the problem. The data for our analysis encompass 23 key informant interviews with national health sector actors working in leadership positions on MNCH in Ethiopia, and six policy documents. Findings were derived from thematic and content analysis.

**Results**: Health inequity is a normalized and inevitable concern that is regarded as actionable (can be altered) but not fully resolvable (can never be fully achieved). Operationally, health equity is viewed as a technocratic matter, reflected in the widespread use of metrics to motivate and measure progress. These representations are shaped by Ethiopia’s rapid expansion of health services into rural areas during the 2000s leading to the positive international attention and funding the country received for improved MNCH indicators. Expanding the coverage and efficiency of health service provision, especially in rural areas, is associated with economic productivity.

**Conclusion**: The metrication of health equity may detract from the fairness, justice, and morality underpinnings of the concept. The findings of this study point to the implications of global pressures in terms of maximizing health investments, and call into question how social, political, and economic determinants of health are addressed through broader development agendas.

## Background

Health equity, long a part of public health policy and practice, has become a formal part of global health policy discussions and a priority across countries [1,2]. Efforts to define, quantify, assess, and promote health equity have proliferated in recent years: health equity is part of global United Nations development agendas, with the Countdown to Equity Working Group prompting a greater focus on the concept during the implementation of the Millennium Development Goals (MDGs), and its more explicit inclusion in the subsequent Sustainable Development Goals (SDGs) [3,4]. Equity is embedded in mission and purpose statements of numerous health and health-adjacent institutions, and is a growing area of academic research across various disciplines [5].

Health equity in the academic literature is generally defined as the absence of systematic differences in health across population subgroups, based on social, demographic, or geographic characteristics that are judged to be unfair and avoidable by reasonable action [6,7]. In contrast to concepts of health inequality or health disparity – which often evoke an objective representation of the distribution of health – health equity is said to be subjected to ethical/moral determinations about distributive fairness and the obligations of governments and other actors [8]. Concepts of human rights and social justice have been widely associated with health equity [9,10]. (For more information about the theoretical concept of health equity, see Supplementary File 1.)

Analyses of health equity that are rooted in a realist epistemology probe a deeper understanding of the processes, reasons, and contextual factors that contribute to (in)equity. These types of analyses focus on building and refining theory about the drivers of health equity, identifying causal mechanisms, and proposing solutions to ameliorate inequities [11]. The assumption that health inequity constitutes a problem (or, to some, a ‘wicked’ problem [12]) motivates responses in health policy spaces; characterization of the problem itself is often left unexplored and unquestioned. Thus, this article addresses the problematization of health equity. Through a case study of maternal, newborn, and child health (MNCH) in Ethiopia, we pose the question: how is the problem of health inequity constituted within policy discourses in Ethiopia? In our analysis, we aim to characterize how health equity is represented as a policy issue, how this representation came about, and the assumptions that underlie it. Our decision to frame health (in)equity as the ‘problem’ reflects an understanding of the concept not as a dichotomy (i.e. equitable vs inequitable) but rather as a continuum (i.e. the extent/degree of health equity).

### MNCH in Ethiopia

During the MDG period (1990–2015), Ethiopia reported national improvements in MNCH indicators, attributed to the expansion of health services in rural areas through the Health Extension Program – progress that garnered positive attention from the international community and instilled a national sense of accomplishment. While the World Health Organization Regional Office for Africa acknowledges that Ethiopia was able ‘to propel its health system to such success’ [13], the country’s own *Health sector transformation plan* describes the progress registered in Ethiopia during the MDG period as a rise-from-behind narrative where ‘Ethiopia’s health indicators have been remarkably improved from one of the worst in Sub-Saharan Africa to amongst the stand-out performers in just two decades’ [14]. For example, in 2015 the maternal mortality rate in Ethiopia (353 deaths per 100,000 live births) was lower than the average rate across Sub-Saharan Africa (547 deaths per 100,000 live births), whereas in 1990, Ethiopia’s rate was much higher (1250 vs. 987 deaths per 100,000 live births in Ethiopia vs. across Sub-Saharan Africa, respectively) [15]. Ethiopia’s 2017 SDG voluntary national review positions the country as an exemplar in global development, noting that the country ‘registered remarkable achievements’ in the MDGs and ‘has made significant contributions by sharing [lessons from the MDG experiences] as inputs to the preparation of the 2030 Global Agenda for Sustainable Development’ [16].

Ethiopia’s achievements in MNCH during the MDG period, however, were not equitably realized by all with, for example, health service coverage varying between urban and rural populations, and between subnational regions [17,18]. In the 2016 Ethiopia Demographic and Health Surveys, the coverage of antenatal care visits in urban areas was substantially higher (90%) than rural areas (58%) and, while nearly all women in Addis Ababa gave birth at a health facility (97%), fewer than 1 in 5 had a health facility birth in the regions of Affar (15%), Somali (19%) and Oromia (19%) [19].

Pursuing equity in MNCH is a priority nationally for Ethiopia in the SDG era, as well as for international donors and development partners. On national stages, countries such as Ethiopia have adopted equity as part of health planning and policy objectives, and the concept of equity is apparent within MNCH programs and policies [17]. Moreover, health equity is part of Ethiopia’s international and national health and development commitments, including the SDGs, the African Union Agenda 2063 and universal health coverage.

## Methods

### Theoretical approach

We undertake a deep evaluation of the problematization of health equity by national-level actors in the Ethiopian health sector. We draw from Bacchi’s analytical approach (‘what is the problem represented to be?’ or WPR approach) [20]. The WPR approach seeks to uncover normative assumptions embedded in policy problems, in this instance ‘health (in)equity,’ that appear obvious and taken for granted. The approach highlights the silences and implications of particular problem representations, offering new insights into how policy responses are constructed, justified, and implemented. The WPR approach has been widely used in health research as a theoretical basis to explore the problematization of topics such as gender mainstreaming [21], drugs [22], alcohol [23], and trade policy [24].

**Figure 1.**
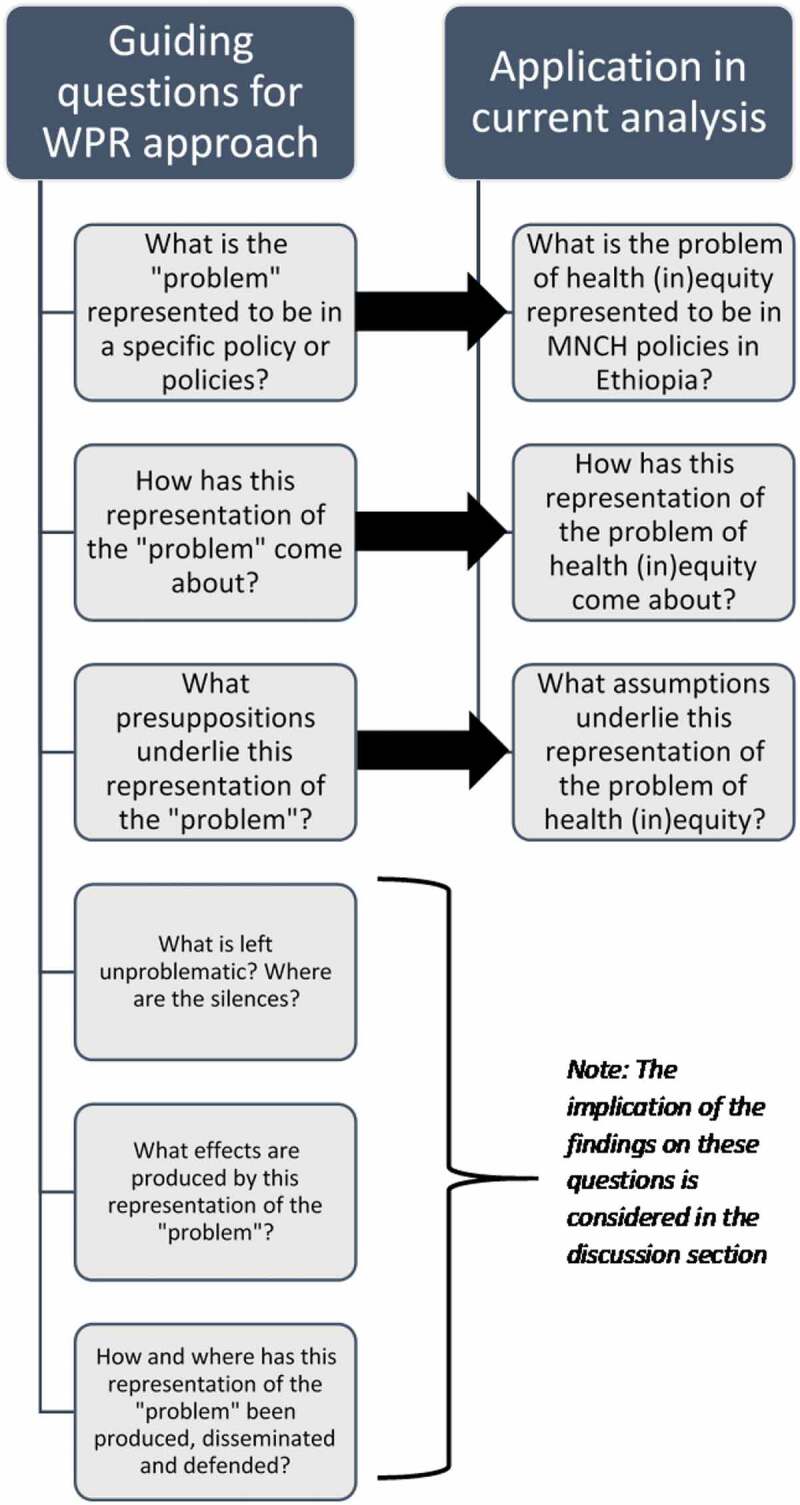
Application of the WPR approach to analyze the problem of health (in)equity in MNCH in Ethiopia

Following the WPR approach, we posit that MNCH policy discourses contain implicit representations of health (in)equity, which can be further understood by analyzing the governing practices and interventions designed to ameliorate it. The guiding questions of the WPR approach, which we use as a heuristic tool, inform our analysis (Figure 1). We present the findings of the study as responses to the first three questions of the WPR approach, which in the current analysis, are formulated as:

1. What is the problem of health (in)equity represented to be in MNCH policies in Ethiopia?
2. How has this representation of the problem of health (in)equity come about?
3. What assumptions underlie this representation of the problem of health (in)equity?

In our application of WPR, we acknowledge that the discourse and knowledge surrounding governance practices extend beyond a sole focus on official policy documents. In the context of Ethiopia, English-language health policy documents draw heavily upon the texts, terminology, and framing used in publications by organizations external to the country. For the purpose of this study, we sought contextualization through direct discourses with policy actors. We present empirical findings from interview data and use document analysis for triangulation purposes. We undertook this study as part of a nested case study that explores perceptions and experiences related to health equity among national, subnational, and community stakeholders in Ethiopia [25–27], conducted as an extension of the Safe Motherhood Project [28]. These prior analyses at the community and subnational levels shaped how the researchers understood the research context and subject matter.

### Data collection and analysis

Our analysis draws from 23 key informant interviews conducted with individuals in Ethiopia working on national MNCH issues in leadership roles across different types of health sector organizations. We identified potential participants purposively based on their professional affiliation and senior position, and interviews were conducted between November 2017 and January 2018. The participants were affiliated with government ministries (n = 5), research institutes (n = 2), academic institutes (n = 5), international organizations (n = 3), donor/implementing organizations (n = 6) and civil society organizations (n = 2), and held titles such as ‘director,’ ‘coordinator,’ ‘senior expert’ and ‘lead researcher.’

Semi-structured interviews covered five domains of questioning: perceptions of relevant social determinants of health^1^^1^Understandings of social determinants of health – the factors that shape the conditions in which people grow, live, work, and age – help to clarify contextual representations of health equity, as actions on the social determinants of health are recommended to tackle situations of health inequity (29).; health equity within current scope of work; interface with health equity work at national and/or global levels; interface with health equity work at subnational levels; and collaborations with other sectors or groups.^2^^2^Intersectoral collaborations between actors in health and non-health sectors contribute to the improvement of health equity (29), and questioning about these collaborations revealed how health equity was represented in these interactions. All interviews were conducted in English, and the duration of the interviews spanned from 30 to 75 minutes. At the request of one invited participant, one interview was done with two participants present (a manager, who was the invited participant, and a trainee working with the organization). One interview was conducted over two sessions, as the participant was called away partway through the initial session. All participants were asked for permission for the interview to be audio recorded. One participant did not give consent to be audio-recorded, and the researcher took notes during the interview, including jotting down exact quotes. All other participants gave consent for the interview to be audio-recorded and transcribed.

To triangulate findings we relied on six selected policy documents, including national strategies, plans, and reports, to represent the major priorities and directions for MNCH and the national health sector. The documents were available in English and current at the time of the research (2017–2019). We obtained ethical approval for the study from the University of Ottawa and Jimma University, and all participants provided written informed consent for their participation in the study.

For more information about the key informant interviews and policy documents, see Supplementary File 2.

We derived the findings from a thematic and content analysis of the interview transcripts and policy documents using Atlas.ti software (version 7.5.18, ATLAS.ti Scientific Software Development GmbH). NB led the analysis, with inputs and guidance from other co-authors who have previous experience with the application of the WPR approach. Researchers did a comprehensive reading of all transcripts and documents to develop an understanding of the discourses and to consider gaps and silences in the texts. Preliminary coding of the interview transcripts identified passages that related to the importance of health equity, and ownership and accountability for health equity (where health equity was considered both broadly and specifically in relation to MNCH topics). We produced coding summaries of the relevant quotes for each code, and rearranged quotes into major thematic categories. The content within the thematic categories was then consolidated as descriptive text, further summarized and, where relevant, grouped to correspond to key questions identified in the WPR approach (pertaining to the representation of the problem, the origins of the representation, and the underlying assumptions). To enrich and triangulate these findings, we analyzed policy documents through a similar approach; that is, identifying relevant passages and then thematic ideas related to: descriptions of health equity; stated importance; evaluation or measures of progress; historical context; and ownership and accountability.

## Results

### What is the problem of health (in)equity represented to be?

#### Normal, inevitable, and actionable

While participants have diverse understandings and perceptions of health equity, the pursuit of health equity as a national health sector priority for MNCH is generally regarded as normalized and inevitable, as actionable (can be altered) but not fully resolvable (can never be fully achieved); hence, a dialectic. The concept of health equity was familiar to all study participants, who readily recognized its prominence in government health plans and strategies, in MNCH and in those focused on health more broadly [14,29,30].^3^^3^Participants tended to speak about the concept of health equity holistically, without specifying between equity in health services versus equity in health outcomes, though cases where this distinction was made are indicated.

In speaking about the national situations in Ethiopia and abroad, participants expressed health equity as a priority for Ethiopia, but also one that exists for other countries. P1 elaborates on this idea, acknowledging that the international acceptance of the importance of improving health equity is an impetus for action:

In terms of [addressing] inequity, the Nordic countries seem to fare better. But we know this is a challenge everywhere. In terms of equity, people [in Ethiopia] feel the gap growing … in that sense, we need to address it. –P1, academic institution

In this quote, P1 expresses an idea also echoed by other participants, alluding to an acceptance of the notion that the meanings or expectations surrounding health equity are pluralistic, and can be adapted based on the country context. Some, including P3 from an academic institute, further assert that even within Ethiopia the expectations associated with health equity may be different. P3 explains their view about the unrealized potential of the health system to equitably deploy health interventions such as vaccination:

If you take this [health equity] as the opportunity that everyone has the fair chance to receive [health care], you don’t expect a country like Ethiopia to provide hospital bed services to everyone. At least you know the availability of health posts in every village, and the provision of very cost-effective interventions like vaccination for everyone – although we have not used the whole potential [of the health system]. –P3, academic institution

Acknowledging perceived lower expectations surrounding Ethiopia but also the potential of the health system to do more, P3 raises an inherent tension between the inevitable-yet-actionable nature of equity concerns. Reconciling the prioritization of health equity within Ethiopia – a country with high universal need and widespread poverty – presents as a complexity for some. While several participants made remarks to the effect of Ethiopia ‘doing well in sharing poverty,’ a few go further by questioning why equity should even be considered a priority in the health sector given the extent of poverty and lack of basic infrastructure such as roads. As P14 explains:

Equity is … a luxury, to be honest with you. [O]ur health facilities are not well equipped, our health personnel are not trained and not well paid. The reality on the ground is very dark […] Without the basics of road infrastructure, without the skilled manpower, talking about equity is a bit far fetched for me. […] if you consider the poverty we are in. –P14, research institute

Decoupling equity from the underlying determinants of health and poverty, P14 suggests that a minimum threshold of development is required before health equity can be taken seriously as a policy priority. This reinforces many interviewees’ sense of the inevitability of inequity, since addressing it first requires improvements in widespread poverty and other conditions that are necessary prerequisites for improved health.

#### A technocratic matter

Discourses surrounding health (in)equity – both in the key informant interviews as well as policy documents – largely represented health equity as a technocratic matter operationalized as the distribution of health intervention coverage [16,31]. As such, (in)equity represents a problem that can be quantified and pursued strategically through a dedicated focus on improving the coverage of priority health services.^4^^4^In the area of MNCH, the country has implemented a set of high-impact services to address maternal mortality, namely, family planning, skilled birth attendance, antenatal care, and postnatal care. Efforts to monitor and promote equity in MNCH center on expanding the coverage of these services. P13, a director at a federal research institute, explains:

So yeah, in Ethiopia, equity is very important in terms of geographic equity in health care … I have seen data that shows where these midwives, skilled midwives, are assigned, skilled attendant deliveries have gone up. Also in those zones where they have beefed up immunization programs to address equity, I’ve seen that immunizations has gone up. –P13, research institute

When reflecting upon health equity successes in MNCH in Ethiopia, participants often cited the Health Extension Program, established in 2003 to expand health service coverage into rural areas. Speaking about future plans to promote health equity, participants conveyed a need to expand service coverage. P16 from the Ministry of Health (MoH) expressed a sense that so-called emerging regions were falling behind, as they work to improve their performance on key indicators:

We are just trying to catch up the other emerging regions so at the end of the day we’ll assess our effort based on the key performance indicators that [are reflected in] our monitoring and evaluation framework of the strategic document. –P16, MoH

According to participants, some non-governmental organizations provide financial incentives for achieving certain MNCH indicator targets, suggesting that technocratic representations of health equity are also reflected in the economic framing of the problem and solution (see following section). P17, from the MoH, outlines how increasing the number of health extension workers (locally recruited, salaried health workers with basic training) from two to three per health post could help to improve coverage of priority MNCH services:

We are planning to increase our numbers of health extension workers … we have recommended three health extension workers at each health post so that they can cover all the areas and [expand health services at that level]. –P17, MoH

### How has this representation come about?

The representations of equity as actionable and quantifiable stems from an overarching framing of MNCH as part of a larger economic development strategy. Growth and Transformation Plan II (GTP-II), which sets the foundation for sectorial plans in Ethiopia, is oriented towards Ethiopia attaining lower middle-income country status by 2025, a development target in many low-income countries in Africa [30]. Accordingly, the health sector frameworks described in the *Health sector transformation plan* were developed to support this vision [14]. Using an analogy of health services as products and health outcomes as profits, P10 from a donor/implementing organization explains how access to MNCH services is a precursor to measuring outcomes, and further highlights the economic trade-offs of investing in subnational regions with variable levels of development:

You start with access. You get the consumer to the facility, you consume the services and then you measure outcome. If you don’t have anyone buying the product, you won’t know how successful you are in marketing the product or you know, in gauging your profit … If you do an investment in Oromia and you impact 50 sites, you will get the numbers you need, versus if you combine Gambella, Benishangul and Somali regions, with 20 times the effort, you still won’t be able to reach that end. –P10, donor/implementing organization

The argument presented by P10, and also expressed by several others, conveys a circularity of health and development whereby economic productivity is viewed as a determinant of health, and investing in health is viewed instrumentally as a means to improve productivity. P10 emphasizes that putting resources into situations with more complex needs will yield lower returns; thus, addressing health (in)equity requires increased investments in health that are disproportionately higher in ‘emerging’ regions. This unveils a tension between framing health for its instrumental purpose (which, according to P10, may exacerbate health inequities) and addressing equity concerns (which may stall development).

The health sector in Ethiopia brings substantial financial capital into the country. The *National strategy for newborn and child survival in Ethiopia* reports that the national health expenditure increased by 138% between 2007/08 and 2010/11, with about half of contributions coming from donors [29]. Ethiopia has attracted external funding based, in part, on its achievements in expanding health coverage to rural areas (i.e. making progress to address equity in MNCH), and continues to receive financial inputs, concurrently motivating both the normalization and the problematization of health equity in policy spaces.

Receiving external donor funding has influenced the positioning and autonomy of the MoH, both internal to the government as well as among international and non-governmental organizations. P22, from an international organization, has experience working in other countries, and explains how the situation in Ethiopia is unique:

I think what is interesting in Ethiopia is that the Ministry of Health is one of the most powerful ministries in government, which is different from other places where I’ve worked … The Ministry of Finance doesn’t have the leverage over the Ministry of Health that it might in other countries. The Ministry of Health has resources, they have a growing public budget and a ton of donors and technical assistance. –P22, international organization

This participant goes on to explain that the MoH in Ethiopia has established this high level of autonomy through a strong track record of ‘producing results’ in line with international donor interests; this participant notes that, from the perspective of external donors, ‘for now we are lucky.’

### What are the underlying assumptions?

The normative representations of health (in)equity as a normalized, inevitable, and actionable national policy problem reflect certain assumptions regarding: responsibilities and roles of stakeholder groups; the connections between health outcomes and economic productivity; and the ability of coverage estimates to reflect reality. Health equity is assumed to be, ultimately, the responsibility of the government. In describing their respective organizations with regards to promoting equity in MNCH and health more generally, participants oriented their roles in alignment with, or as filling the gaps of, the work of the MoH (Table 1). Recognition for advancing equity is primarily directed towards the government and, under the purview of the government, health equity has been mainstreamed and legitimized (i.e. normalized) across health sector discourses and planning more broadly. For example, P7, from a civil society organization, describes their role in ensuring that the government follows through on its responsibilities for equity:

As a civil society organization, we are concerned about equity, but we are not responsible for equity. The government is responsible for equity. But we are responsible to impose the government so that health equity comes. –P7, civil society organization

**Table 1.** Major roles in promoting health equity, self-described by national health sector stakeholders

Stakeholder group	Major roles in promoting health equity
Ministry of Health	-technical and advisory role for Regional Health Bureaus -advocacy -leadership and coordination among other stakeholder groups -special support to low-performing areas (e.g. through mentorship initiatives, training, supervisions, funding)
Research institute	-gather evidence to inform decision making by the Ministry of Health -promote knowledge translation and uptake of evidence for decision making
Donor/implementing organization	-work across levels of the health system in a variety of roles including program delivery, system strengthening, advocacy, financing and others -address needs that are unmet by the government health system (‘gap filling’) -often have expertise about realities at the community level
Civil society organizations	-advocacy and information dissemination, both with the government and their members and networks -fulfill a coordinating role, such as hosting policy dialogues and forums
International organizations	- technical and financial support to the Ministry of Health, including assisting with the preparation of government strategies and implementation of global standards -maintain databases and build capacity in data management -convening role among development and implementing partners
Academic institute	-engage with health equity issues through projects, research and training -facilitate community-based learning experiences for students -participate in government research advisory councils -produce policy briefs, systematic reviews and meta analyses for government policy makers

Caption: This table shows the self-described roles of the stakeholder groups, derived from key informant interviews and policy documents.

The MoH, with positive reinforcement on the international stage for their recent accomplishments, support from various health sector groups in the country, and a fairly autonomous leadership position within the wider government, is highly incentivized to exert ownership over health equity.

Embedded in national discourses is an assumption that health equity is considered more actionable (and perhaps even resolvable) at decentralized levels of the health system. An overarching focus on resource efficiency means that decentralized levels of the system are called upon to report improvements in health service use without substantially increasing resource inputs, but rather through innovation and ingenuity. National actors emphasized the importance of community ownership and action over MNCH initiatives, with P18 from the MoH noting that ‘you’d be surprised what the community does in different places.’ Participants from the MoH mentioned several low-resource ways that their organizations encouraged innovation and improvement at decentralized levels of the health system, such as rewarding high-performance, facilitating best-practice sharing, and implementing mentorship programs. Several participants from donor/implementing organizations also described a reliance on inbuilt community potential, evident through a trend of moving their resources ‘out of the community’ to instead focus on health system strengthening at the district level and above. P10 explains how their donor/implementation organization is shifting support out of the community:

Unless you pull up [to support higher levels of the health system] and you start creating capacity to eventually hand over to the true owner of the system, you continuously will be doing the work for them, and not building any capacity and transitioning this work for them. –P10, donor/implementing organization

The pro-poor and pro-development aims of GTP-II are used to justify the prioritization of health equity – more specifically, promoting universal access to MNCH services – in health policies and reports, reiterating the circularity of health and development. This notion reflects an implicit assumption that: (a) the use of health services will (b) improve health outcomes and, (c) in turn facilitate economic development. These links (i.e. that (a) leads to (b) and that (b) leads to (c)) are further associated with the pursuit of health equity through normative statements such as this passage from the *State of equity* report:

Equity in accessing health services and health outcome has been recognized as an important development agenda for Ethiopia as it would be a concern for further development due to a potential widening in economic growth. [32,p.73]

Commonly, health equity discourses encountered in this research made direct links between health service provision and economic productivity, implying that health service indicators may be a proxy for health status. Accordingly, much emphasis is on rapidly maximizing the coverage and efficiency of health service provision as a means to drive economic development. This calls into question the extent to which the idea of leaving no one behind is attainable: while the government states a position promoting equitable health service access and health outcomes, external financing seeks to optimize economic returns on investments in health. As P18 explains, this also introduces concerns surrounding quality and sustainability:

Trying to improve access has been a main focus of the programs and development partners and the government as well. Through that process, I think, quality has been compromised. Mainly because we are trying to meet numbers and ratios of the health care professionals, ratios of the facilities and the like. In the meantime, we have compromised quantity for quality. Quantity and quality should have gone hand in hand, but we are a resource-limited nation and we had to do what we had to do. –P18, Ministry of Women and Child Affairs

P18’s resigned acceptance of the perceived compromise between quantity and quality begs greater consideration of the consequences of striving to meet numbers and targets.

With regards to the representation of health equity as a technocratic matter, the use of metrics to motivate and measure the advancement of equity rests on the assumption that these metrics are – or have the potential to be – an accurate reflection of reality. In Ethiopia, the implications related to the metrics surrounding MNCH service coverage and outcomes are reflected in funding levels and arrangements, national and international positioning of health sector organizations, and recognition and classification. Key informants raised widespread concerns surrounding the integrity of health information systems and expressed a desire to improve these systems – for example, by introducing more rigorous auditing procedures or data collection practices.

## Discussion

The findings of this study underscore that, while health equity may appear to be a universal and unquestionable concept, it may actually be highly contested and open to multiple translations and meanings [33,34]. In the context of our analysis, the pursuit of health equity as a policy priority is portrayed as normalized and inevitable in the health sector. In this representation, the advancement of equity becomes a permanent ‘work in progress’ where the problem of health inequity can continually be redefined and, in effect, never truly resolved. Accordingly, Ethiopia is legitimized in keeping equity as part of policy discourses. This representation of health equity alludes to the idea of progressive realization, whereby countries move at their own pace to progress towards full realization of a health goal, given the availability of resources.

The conflation of health equity and health service equity (which is the basis of much confusion surrounding the meaning of health equity) provides a certain advantage in policy spaces, as addressing inequalities in health services is a concrete policy aim [35]. Defining equity through such metrics provides a clear mechanism within the health sector for the coordination of action by multiple stakeholders. It also serves as a way to maintain administrative oversight and hold stakeholders accountable, reinforcing implicit hierarchical power relations between funders and funding recipients as well as actors at different levels of the monitoring and evaluation process. A study of a pro-poor initiative to increase health service use in Cambodia acknowledged the importance of accounting for the quality of services, and prevailing social environment of health facilities, such as hospital mission statements, alongside efforts to increase coverage [36]

The findings of this study surrounding the emphasis on economic development have implications for MNCH policy beyond Ethiopia, as low- and middle-income countries face similar global pressures in terms of maximizing health investments. By focusing on economic development, wider civil liberties and political freedoms may be effectively de-emphasized. In India, for example, the Rashtriya Swasthya Bima Yojana health insurance program for people below the poverty line has been characterized as a ‘social investment in economic growth’ with a major interest in improving worker productivity and economic development [37]. When economic interests become the primary drivers for action, human development motives, such as those espoused by the 2030 Agenda for Sustainable Development, are at jeopardy of being sidelined. Ethiopian-based scholars suggest that broader civil engagement and expanded democratic spaces are required to generate wider support and ownership over health reforms, and thereby advance development aspirations [38].

While issues in Ethiopia surrounding interracial violence, forced displacement, human rights abuses, political oppression, and gender-based inequality have been highlighted in international media and reports [39–42], they remain outside of the bounds of health equity metrics and are only rarely raised in health research [43]. The application of a meaningful legal and policy frameworks to realize and uphold human rights, some argue, is part of a way forward in promoting health equity and legitimizing democratic health reforms [44].

The global dimensions of health equity, driven by macro-structural transformations and global policy priorities, are left largely unexplored in policy documents and by key informants. Ethiopian studies on this topic have tended to bracket health outcomes, focusing on the positive growth impacts of trade, global market openness, and foreign investment [45,46]. Others are more critical of the country’s reliance on foreign investment, growing foreign debt, and emphasis on export-processing zones known for low wages and oppressive working conditions [47,48], and there is generally little reference to health impacts either positive or negative. However, deep inequities in the distribution of power and economic arrangements, globally, are of key relevance to understanding health equities in national and local contexts [49]. For example, structural adjustment programs administered by international financial institutions have been found to contribute to growing health and income inequality in African countries [50,51], while policy reforms driven by the World Bank, especially the privatization of health services through pubic private partnerships, have undermined equitable access to health-care provision [52]. The importance of such global policy choices was not reflected in the dominant representations of health equity discourse in Ethiopia, depoliticizing the role of global social relations and their complicity in reproducing an unjust economic order leading to inequitable health outcomes.

Aspects of the political landscape in Ethiopia variably reinforce and challenge the conditions that perpetuate current representations of health equity. Ethiopia has a poor human rights record that does not reflect the rights and freedoms guaranteed in its constitution or the numerous international human rights agreements to which it is a signatory [53]. Despite its ambition to achieve lower middle-income country status by 2025, large segments of the population still lack access to basic infrastructure and social services. These realities uphold the notion that there are persistent imbalances of power, resources, and wealth in the country that systematically disadvantage certain groups within the population, reinforcing the inevitability of inequities, including in health. On the other hand, the change of government in April 2018 that saw Dr Abiy Ahmed become prime minister initially brought renewed optimism for political and economic reforms that, to date, have been met with mixed success (marred by sporadic internet outages, escalating ethnic tensions, ongoing land conflicts, and large numbers of internally displaced peoples, among other issues).

### Strengths and limitations

Drawing from a WPR approach, this study offered new insights into understanding the problematization of health equity in Ethiopia. This research was strengthened by the inclusion of participants from diverse health-related organizations in Ethiopia, encompassing both governmental and non-governmental organizations. While all participants were currently working on MNCH issues on a national level, many had career trajectories that had spanned several organization types and positions, both at the national level and, in some cases, including practitioner positions at lower levels of the health system. This allowed several participants to speak to varying perspectives across organizations. We acknowledge the small number of participants included in the study, as well as the limited selection of policy documents, and caution that our aim was not to generalize the findings, but rather to provide exploratory insights into the research questions. Thus, we sought to include sufficient depth of information and context to give a sense of the transferability. We recognize the possibility of social desirability bias, and have taken steps to account for and minimize this bias [54].

We did not recruit participants from the private sector in this study. The private sector currently occupies only a small role in the health sector, though the prospect of expanding public–private partnerships is of growing interest to the MoH [55,56]. Thus, the influence of private interests in health equity spaces is an area for future exploration. We note that changing political dynamics in Ethiopia since the time of data collection may have implications for understandings of health equity in the country.

## Conclusion

This article brings together representations of the problem of health inequity in MNCH policy in Ethiopia. Our analysis indicates that health inequity in this context is characterized as an ongoing, technocratic problem, primarily operationalized through the continual expansion of health interventions into rural areas. These representations of health equity depoliticize the problem, turning attention towards improvements in quantifiable health measures and data systems. Discussions of health equity are often synonymous with those about health inequalities or disparities (systematic differences in health across population groups) rather than the acceptability, or unacceptability of measurable health differences under different circumstances. The current representations of health equity may drive the expansion of health service coverage; however, they do little to challenge entrenched power structures, and may detract from more controversial issues such as empowerment, access, participation, and rights. Building on the findings of this study, further research is warranted to delve into the silences inherent in the current representation of health equity, including studying the processes and conditions of how these silences may be brought into health equity policies.

## Supplementary Material

Supplemental Material

## Paper context

While equity in maternal, newborn, and child health is widely accepted as a key health policy priority in Ethiopia, deeper contextualization of how the issue is understood and characterized is lacking. This paper uses Bacchi’s ‘what’s the problem represented to be’ approach as a heuristic tool to explore the normative assumptions embedded in the characterization of health equity in the Ethiopian health sector. The findings yield insight into national and global influences that shape how health equity is addressed.
