# Supplementary material for: Characterizing ‘health equity’ as a national health sector priority for maternal, newborn, and child health in Ethiopia
Source: Glob Health Action. 2020 Dec 30;14(1):1853386. doi: 10.1080/16549716.2020.1853386 (PMC7782227; doi:10.1080/16549716.2020.1853386)
Supplement: Supplemental Material [file ZGHA_A_1853386_SM5348.zip › Supplementary/Supplementary File 1.docx]

# Supplementary File 1

## Constructs of health equity

Health equity can be broken down into the following key constructs: conceptualization of health; distribution of health; and moral or ethical characterization of health distribution (Figure SF1.1). Health actors’ understanding of health equity can be framed around each of the three constructs in turn, addressing first: what aspect of health is given consideration? And how is it described for individuals and/or groups of individuals?

Figure SF1.1. Constructs of health equity [1]

The conceptualization of health may focus on subjective or objective assessments of health status, well-being or functioning [2,3]. It may also capture any other aspect of the health system (inputs and processes, outputs, outcomes and impact), including health governance, health financing, health services access and readiness, and health service coverage [4,5]. Broadly defined, concepts of health may encompass health-related norms, values, behaviours and attitudes, as well as social determinants of health (i.e. social conditions that are inextricably linked with health) [6].

The distribution construct then addresses: how is health distributed between groups of individuals defined by one or more common characteristic^[[1]](#footnote-1)^? Implicit in this construct is the question of how subgroups of individuals are defined and determined [10]. This construct introduces considerations surrounding the comparison of health across subgroups and the assumptions that underlie the approaches and interpretations that lead to certain conclusions [11–14]. For instance, measures that make relative versus absolute comparisons implicitly endorse a position of strict egalitarianism (relative measures) or egalitarianism alongside other considerations, such as level of health (absolute measures) [12].

The third construct addresses whether a specified aspect of health and its distribution is problematic from a moral/ethical perspective. That is: is the distribution of health fair, i.e. are the health differences between individuals or groups unavoidable? [6] This determination is subject to different interpretations within particular contexts, for example, arising from historical or cultural experiences that inform how societies understand notions of fairness. This construct is the normative component central to health equity (which can be distinguished from health inequality, which pertains to the distributional construct) [11]. Moral and ethical judgements about health distribution are a reflection of societal values, as expressed, in part, through social arrangements and obligations of governments [15]. These judgements and values, however, are pluralistic [16]. Labonté (2013) elaborated on the two theorized axes of general values that exist within individuals, the so-called freedom/equality divide: while individualism emphasizes freedoms and national strength/order, communalism emphasizes equality and international harmony. While individuals may espouse values from both axes, the prominence of one axis over the other will likely emerge in accordance with previous individual experiences and influences [17]. Similarly, competing utilitarian and egalitarian perspectives favour the maximization of aggregate health or the maximization of the equal distribution of health, respectively. Taken to extreme, a utilitarian approach may justify the advancement of the well-off group and abandonment of disadvantaged minorities, and an egalitarian approach may result in poor – though more equal – health for everyone [14].

Examinations of health equity lead to the question of: what is to be done (and by whom) about health inequities? Efforts to reduce health inequities might engender tensions such as the reconciliation of equity and efficiency: while equity interests are focused on gains in disadvantaged subgroups, efficiency interests focus on population-wide gains. A second tension is the orientation of remedial actions towards broad contextual factors (e.g. structural aspects of the political economy) versus proximal factors (e.g. specific aspects of the health system functioning). Often with considerable overlap, such factors can be aptly depicted using ecological models, which are useful for visualizing barriers and enablers across domains of influence and generating insights into possible entry points and synergies to improve health. Ultimately, there is consensus about the need for multi-level action to facilitate equity gain [18,19].

References

[1] Bergen N, Ruckert A, Kulkarni MA, et al. Subnational health management and the advancement of health equity: a case study of Ethiopia. Global Health Research and Policy. 2019;4:12.

[2] Fedoryka K. Health as a normative concept: towards a new conceptual framework. Journal of Medicine and Philosophy. 1997;22:143–160.

[3] Sen A. Health equity: perspectives, measurability, and criteria. In: Evans T, Whitehead M, Diderichsen F, et al., editors. Challenging inequities in health: From ethics to action. New York: Oxford University Press; 2001. p. 69–75.

[4] Whitehead M. The concepts and principles of equity and health. Health promotion international. 1991;6:217–228.

[5] World Health Organization. Monitoring, evaluation and review of national health strategies: a country-led platform for information and accountability. Geneva: World Health Organization; 2011.

[6] Dahlgren G, Whitehead M. Policies and strategies to promote social equity in health. Stockholm: Institute for future studies; 1991.

[7] Murray CJ, Gakidou EE, Frenk J. Health inequalities and social group differences: what should we measure? Bulletin of the World Health Organization. 1999;77:537–543.

[8] Gakidou E, King G. Measuring total health inequality: adding individual variation to group-level differences. International journal for equity in health. 2002;1:3.

[9] Braveman P, Krieger N, Lynch J. Health inequalities and social inequalities in health. Bulletin of the World Health Organization. 2000;78:232–235.

[10] Hosseinpoor AR, Bergen N, Schlotheuber A, et al. National health inequality monitoring: current challenges and opportunities. Global Health Action. 2018;11.

[11] Kawachi I, Subramanian SV, Almeida-Filho N. A glossary for health inequalities. Journal of epidemiology and community health. 2002;56:647–652.

[12] Harper S, King NB, Meersman SC, et al. Implicit value judgments in the measurement of health inequalities. Milbank Quarterly. 2010;88:4–29.

[13] Harper S, Lynch J. Methods for measuring cancer disparities: using data relevant to healthy people 2010 cancer-related objectives. 2005;

[14] Alonge O, Peters DH. Utility and limitations of measures of health inequities: a theoretical perspective. Global Health Action. 2015;8:27591.

[15] Braveman PA. Monitoring equity in health and healthcare: a conceptual framework. Journal of health, population and nutrition. 2003;181–192.

[16] Asada Y. A framework for measuring health inequity. Journal of epidemiology and community health. 2005;59:700–705.

[17] Labonte R. Human rights and equity: the value base of global health diplomacy. In: Kickbusch I, Lister G, Told M, et al., editors. Global health diplomacy: concepts, issues, actors, instruments, fora and cases. New York: Springer; 2013. p. 89–105.

[18] Solar O, Irwin A. A conceptual framework for action on the social determinants of health: social determinants of health discussion paper 2 (policy and practice). Geneva: World Health Organization; 2010.

[19] Commission on Social Determinants of Health. Closing the gap in a generation: health equity through action on the social determinants of health: final report of the commission on social determinants of health. 2008;

1. An alternative approach to conceptualizing health distribution is an ungrouped approach, proposed in the late 1990s, which measures the distribution of health among individuals of a population regardless of social groupings (e.g. using measures such as standard deviation or variance) [7,8]. This approach, which borrows from economic theories of income inequality, has been criticized for rejecting the premise that health inequality is linked to social disadvantage [9]. [↑](#footnote-ref-1)
