# Supplementary material for: Characterizing ‘health equity’ as a national health sector priority for maternal, newborn, and child health in Ethiopia
Source: Glob Health Action. 2020 Dec 30;14(1):1853386. doi: 10.1080/16549716.2020.1853386 (PMC7782227; doi:10.1080/16549716.2020.1853386)
Supplement: Supplemental Material [file ZGHA_A_1853386_SM5348.zip › Supplementary/Supplementary File 2-clean.docx]

# Supplementary file 2

## Key informant interviews

Key informant interviews were conducted with 23 individuals (Table SF2.1).  A sample interview guide appears in Box SF2.1.

Table SF2.1. Characteristics of key informants

| Organization type | Participant job titles | Number of participants (males/females) |
| --- | --- | --- |
| Government ministry | Coordinator, Director, Senior Expert | 5 (4/1) |
| Research institute | Director, Lead Researcher | 2 (2/0) |
| Academic institute | Dean, Director, Professor | 5 (4/1) |
| International organizations | Coordinator, Specialist | 3 (2/1) |
| Donor/implementing organization | Coordinator, Director, Health Lead, Manager | 6 (4/2) |
| Civil society organization | Director | 2 (2/0) |

Caption: This table describes the 23 key informants who participated in the study, categorized according to their organization type, and specifying their job titles and male/female ratios.

Note: The organizations represented in this study encompassed: two government ministries (the Ministry of Health (three directorates) and Ministry of Women and Child Affairs (one directorate)), two Ethiopian research institutes; three Ethiopian academic institutions (one academic participant was retired), two United Nations organizations, five donor/implementing organizations (four international with Ethiopian chapters, and one non-religious national) and two Ethiopian civil society organizations.

Box SF2.1. Sample interview guide

## Introduction

Please tell me about yourself and your role at your organization.

*Prompts: education, country/region of origin, position, years in position, main responsibilities in position*

What aspects of maternal, newborn and child health are part of your work?

*Prompts: policy, programs, practices, research*

## Domain 1. Perceptions of relevant social determinants of health

*[Show photo card to informant]*

- 1. Would you please describe what you see in the photo?

*Prompts: What are each of the people in the photo doing? Where might this take place? How common is it? Level of acceptability?*

1. What underlying factors might have contributed to what you see in this photo?

*Prompts: Individual attributes, family and peer influences, health services, community context, governance and policies, culture and social values*

- 1. Are there any determinants of health that come to mind when looking at this scene?
  2. Thinking about your work, do you directly or indirectly have an impact on scenes like the one in the photo? If so, how?

## Domain 2. Health equity within current scope of work

We are going to now turn to talking about the concept of health equity, which you may know, has gained attention through the Sustainable Development Goals.

1. What does the concept of health equity mean to you?

*[Show the informant a card with the WHO definition of health equity]*

1. This is one definition of health equity that has been proposed. What is your impression of this definition?

*Prompt: Does it capture what you have expressed? Anything that you disagree with? Anything to add?*

1. In what ways, if any, is health equity is taken into account within your work in the area of maternal, newborn and child health? (If not, why not?)

*Prompts: policies, programs, practices; social determinants of health across different levels (refer to those mentioned in domain 1, if appropriate)*

1. What kinds of evidence or knowledge do you draw from to guide this work?

*Prompts: data sources, consultations, experts, resources*

1. What do you hope to accomplish, in terms of reducing health inequity in maternal, newborn and child health?

*Prompts: goals, targets; focus on the worst off, reducing gap or gradient approach*

1. How do you know if this work is having the intended impact?

*Prompts: measurement, monitoring*

1. What more, if anything, could be done to promote health equity through your scope of work?

*Prompts: resources (financial, human), political support, technical knowledge, collaborations*

## Domain 3. Interface with health equity work at the global/national level

On the same theme of reducing health inequity in maternal, newborn and child health, I would like to now talk about work that is being done at the global and national levels. Specifically, I would like to learn more about how your work might interface with other efforts to reduce health inequity in maternal, newborn and child health.

1. Can you please tell me about the work that you are aware of at the global/national level in the area of maternal, newborn and child health, and the reduction of health inequity?

*Prompt: important global/national actors; key policies, programs, practices; Sustainable Development Goal Agenda*

1. How do policies or programs at the global level affect your work?

*Prompts: positive/supporting, negative/barriers, resources, political/popular support*

1. What involvement had you have working with others at the global/national level?

*Prompt: consultations, training, providing data/feedback*

*🡪* What were the positive aspects of these experiences?

🡪What were the negative aspects of these experiences?

1. Are there ways that your work around the reduction of health inequity in maternal, newborn and child health could benefit from greater collaboration with actors at the global/national level? If so, how?

## Domain 4. Interface with health equity work at subnational levels

Next I’d like to discuss work that is being done at more local levels, across regions, zones, districts, and communities to reduce health inequity in maternal, newborn and child health, and how your work fits with theirs.

1. Can you please tell me about the work that you are aware of at the regional level in the area of maternal, newborn and child health, and the reduction of health inequity?

*Prompts: important actors; key policies, programs, practices*

1. What work are you aware of at other levels?

*Prompts: zonal level, district level, level of the primary health care unit, and community level; important actors; key policies, programs and practices*

1. How do national policies or programs affect this more decentralized work?

*Prompts: agenda setting, positive/supporting, negative/barriers, resources*

1. In what ways, if any, does work around health equity at subnational levels impact national planning?
2. What involvement had you have working with others at more decentralized levels (regional, zonal, etc.)?

*Prompts: consultations, training, providing data/feedback, coordinating role across levels(?)*

*🡪* What were the positive aspects of these experiences?

🡪 What were the negative aspects of these experiences?

1. Are there ways that your work around the reduction of health inequity in maternal, newborn and child health could benefit from greater collaboration with actors at the regional (etc.) levels? If so, how?

## Domain 5. Collaborations with other sectors or groups

We are now moving into our final set of questions. We have talked about working with others at the global and national levels, and those at more decentralized levels of government. Now I’d like to talk about other groups of stakeholders that play a role in reducing health inequity in maternal, newborn and child health, even if they play a minor part.

1. Who else do you see as playing a role in reducing health inequity in maternal, newborn and child health?

*Prompt: other sectors of government, UN agencies, civil society organizations, bilateral organizations, donors/NGOs, academia*

1. What work are you aware of that is currently being done by these groups that contributes to reducing health inequity in MNCH?

🡪 In what ways has this work been successful?

🡪 In what ways has this work not succeeded?

🡪 How could this work be more successful?

1. Do any of these groups have bearing on your scope of work? If so, what does this look like?
2. Would you work benefit from greater collaboration with any of these groups?

🡪 If so, which groups? What capacity?

🡪 What would be needed for this to happen?

## Wrap up

Thank you for your time. I have now asked all of my questions. Do you have any final thoughts about our discussion today, or other aspects of health equity that you would like to share before we close?

## Policy documents

Overall, the analysis included six policy documents (Table SF2.2). The *Growth and transformation plan II* (GTP-II) serves as the overarching development plan for the country and the basis for the *Health sector transformation plan* [2]. The *Health sector transformation plan* guides all activities in the health sector over the five-year period between 2015-16 and 2019-20 [3], whereas the *National strategy for newborn and chid survival* is specific to MNCH activities [4]. The remaining three documents include a national plan of action to address low-performing regional states and zones [5], the 2017 voluntary national review on the SDGs [6], and a national report on the state of equity in MNCH [7].

Table SF2.2. Policy documents

| Issuing body | Name of document |
| --- | --- |
| Federal Democratic Republic of Ethiopia | Growth and transformation plan II (GTP-II) (2015/16-2019/20) |
| Federal Ministry of Health | Health sector transformation plan 2015/16-2019/20 |
| Federal Ministry of Health | National strategy for newborn and child survival in Ethiopia  2015/16-2019/20 |
| Federal Ministry of Health | Transforming health status and health systems in the developing regional states and selected zones with suboptimal performance: Plan of action, 2016-2020 |
| National Planning Commission | The 2017 voluntary national reviews on SDGs of Ethiopia: Government commitments, national ownership and performance trends |
| Federal Ministry of Health | State of equity in maternal, child and reproductive health in Ethiopia |

Caption: This table lists the six policy documents analyzed as part of the study, including the issuing body and the name of the document.

## References

[1] Commission on Social Determinants of Health. Closing the gap in a generation: health equity through action on the social determinants of health: final report of the commission on social determinants of health. 2008;

[2] National Planning Commission. Growth and Transformation Plan II (GTP II) (2015/16-2019/20). Addis Ababa: Federal Democratic Republic of Ethiopia; 2016.

[3] Ethiopia Federal Ministry of Health. Health Sector Transformation Plan 2015/15-2019/20. Addis Ababa: Ethiopia Federal Ministry of Health; 2015.

[4] Ethiopia Federal Ministry of Health. National Strategy for Newborn and Child Survival in Ethiopia: 2015/16-2019/20. Addis Ababa: Ethiopia Federal Ministry of Health; 2015.

[5] Federal Ministry of Health Ethiopia. Transforming health status and health systems in the developing regional states and selected zones with suboptimal performance: Plan of action, 2016-2020. Addis Ababa: Federal Ministry of Health Ethiopia; 2016.

[6] National Planning Commission. The 2017 Voluntary National Reviews on SDGs of Ethiopia: government commitments, national ownership and performance trends. Addis Ababa: National Planning Commission; 2017.

[7] Ethiopia Federal Ministry of Health. State of equity in maternal, child and reproductive health in Ethiopia. Addis Ababa: Ethiopia Federal Ministry of Health; 2018.
